# Mitigation Strategies for Human–Tibetan Brown Bear (*Ursus arctos pruinosus*) Conflicts in the Hinterland of the Qinghai-Tibetan Plateau

**DOI:** 10.3390/ani12111422

**Published:** 2022-05-31

**Authors:** Yunchuan Dai, Yi Li, Yadong Xue, Charlotte E. Hacker, Chunyan Li, Babar Zahoor, Yang Liu, Diqiang Li, Dayong Li

**Affiliations:** 1 Institute for Ecology and Environmental Resources, Chongqing Academy of Social Sciences, Chongqing 400020, China; yunchuan.dai.chn@gmail.com (Y.D.); lcyqq1980@126.com (C.L.); 2 Research Center for Ecological Security and Green Development, Chongqing Academy of Social Sciences, Chongqing 400020, China; 3 Research Center for Economy of Upper Reaches of the Yangtze River, Chongqing Technology and Business University, Chongqing 400067, China; cq_liyi@163.com; 4 Key Laboratory of Biodiversity Conservation, State Forestry and Grassland Administration, Ecology and Nature Conservation Institute, Chinese Academy of Forestry, Beijing 100091, China; xueyadong334@163.com; 5 Department of Biological Sciences, Duquesne University, Pittsburgh, PA 15282, USA; hackerc@duq.edu; 6 State Key Joint Laboratory of Environment Simulation and Pollution Control, School of Environment, Tsinghua University, Beijing 100084, China; goldensnow786@gmail.com; 7 School of Tourism, Kaili University, Kaili 556000, China; liuyangliujin1992@163.com; 8 Key Laboratory of Southwest China Wildlife Resources Conservation (Ministry of Education), China West Normal University, Nanchong 637009, China

**Keywords:** human–wildlife conflicts, coexistence, effective measures, Sanjiangyuan region, China

## Abstract

**Simple Summary:**

The conservation of Tibetan brown bear (*Ursus arctos pruinosus*) and its habitat is of great value to the conservation of sympatric species, which helps to maintain the health and stability of the regional ecosystem. In recent years, human–bear conflicts (HBCs) have intensified in the Sanjiangyuan Region in Qinghai Province, China, decreasing the tolerance of local herders of the species and seriously affecting the motivation of local communities to protect brown bears and other wildlife, with retaliatory killing posing a threat to their survival. Timely development of effective measures and countermeasures for mitigating HBCs is crucial to protect brown bears. The mitigation or resolution of HBC issues is beneficial to both the promotion of people’s livelihoods and the conservation of brown bears on the Qinghai-Tibetan Plateau (QTP). At present, there is still a lack of research on the mitigation measures of HBCs on the QTP. This study combined field surveys, semi-structured interviews, and HBC seminars to understand the effectiveness of current mitigation measures and to propose potential mitigation measures in the hinterland of the QTP. This work proposed targeted mitigation measures for HBCs taking into account existing HBC management practices in China and abroad, and the unique geographical environment, laws and regulations, folk culture, and religious beliefs of local regions. Although this study was limited to a single species on the QTP, the results herein are useful for drafting national-level wildlife conservation policies, compensation programs for wildlife damage, and natural resource conservation regulations.

**Abstract:**

Personal injury and property damage caused by wildlife can worsen the relationship between humans and wildlife. In recent years, conflicts between herders and Tibetan brown bears (*Ursus arctos pruinosus*) (human–bear conflicts; HBCs) on the Qinghai-Tibetan Plateau have increased dramatically, severely affecting community motivation for the conservation of brown bears and other species. Understanding the types, effectiveness, and flaws of current HBC mitigation measures is critical to develop effective strategies to alleviate HBC. From 2017 to 2019, we conducted a systematic field survey regarding HBCs on the Qinghai-Tibetan Plateau. In addition, we invited bear specialists and multiple interest groups to hold an HBC seminar and proposed some potential mitigation strategies. We surveyed 312 families via semi-structured interviews and documented 16 types of HBC mitigation measures. A total of 96% of respondents were using more than two mitigation measures simultaneously. The effectiveness evaluation of HBC mitigation measures showed that: (1) removing food from winter homes while herders were at their summer pastures and asking people to keep watch of winter homes were effective at protecting food and houses; (2) traditional grazing methods (human guarding of livestock all day) and solar soundboxes (attached to livestock) were effective at protecting free-range livestock; (3) solar street lights had a deterrent effect on brown bears and were effective in protecting livestock, houses, and people; and (4) due to the unstable power supply of photovoltaic cells and improper installation of ground wires, electric fences were not ideal in practice. Evaluation of the potential mitigation measures at the seminar showed that upgrading electric fence technology, expanding electric fence pilot areas, installing diversionary feeders, and introducing bear spray were the most optimal solutions. This study provides a scientific basis for creating human–bear coexistence plans on the Qinghai-Tibet Plateau.

## 1. Introduction

Human–wildlife conflicts (HWCs) are direct or indirect interactions that result in negative outcomes for humans and/or wildlife [1,2,3]. HWCs are influenced by multiple factors, including complex social, economic, political, religious, and ecological contexts [4,5,6,7,8]. Studies regarding HWCs have been conducted on conflict characteristics, behavioral changes in the species involved [1], interests driving the conflicts [9], and the tolerance of local communities of wildlife [10,11]. Mammals cause the most serious damage to human life and property, particularly species from the families Felidae, Canidae, Ursidae, and Suidae [8,12,13]. The most typical types of HWCs include crop trampling, livestock predation, home invasions, zoonotic disease transmission, and personal injury [5,6,14,15,16,17].

The geographic distribution of different bear families varies, as do the causes of human–bear conflicts (HBCs) [5,6,14]. In North America, grizzly bears (*Ursus arctos horribilis*) and black bears (*U. americanus*) damage human properties due to increasing bear populations and seasonal shortages of natural food resources [18,19]. In eastern Europe, HBCs have been linked to a decline in public tolerance of brown bears [20,21,22]. In Asia, HBCs are associated with the recovery of bear populations, the expansion of human activity, habitat loss, changes in human lifestyles, and changes in bear foraging behavior [1,23,24]. Generally speaking, the main factors leading to increases in HBCs include the recovery of wildlife populations, changes in human behavior, the expansion of anthropogenic activities, increase of livestock, change of land use types, loss of wildlife habitat, and global climate change [25,26,27,28,29,30,31].

In recent years, Western countries have carried out a plethora of research on control measures for mitigating HBCs. Among them, one of the representative achievements is the HBC Management Plan (HBCMP), which contains a series of supporting measures, such as electronic fences, steel boxes, bear spray, and relocation of bears [22,32,33]. In 2009, Canada included the use of steel boxes in the Whistler HBC Management Plan and promoted it in Vancouver and other HBC hot spots in the country [33]. The U.S. Fish and Wildlife Service has advocated that wildlife management staff should be equipped with bear spray when patrolling in the wild. Bear spray is an effective bear deterrent and can correct their behavior of harming people [32,34]. The U.S. Fish and Wildlife Service in Washington mitigated HBCs by forcibly relocating bears that frequently wandered near community dump sites [35]. The U.S. Department of Agriculture (USDA) and the Natural Resources Defense Council (NRDC) set up electric fences in many orchards, which successfully stopped the invasion of American black bears. In addition, high-voltage pulses associated with electric fences contributed to behavioral change in the bears’ intrusion behavior [22,32]. Legal hunting is also an important method of controlling bear populations and mitigating HBCs in Western countries. Wildlife management agencies hunt a certain percentage of adult female bears during the non-breeding season to mitigate HBCs [36]. Male bears also serve as popular hunting trophies. Legalizing hunting may increase public acceptance of bears, thus benefiting their long-term conservation [37]. In the United States and Canada, bears can be legally hunted by the public with a hunting license during open season (non-breeding season). Up to 50,000 bears are legally harvested each year, successfully controlling the bear population and reducing the frequency of HBCs (Black Bear Legal Status & Management; https://westernwildlife.org/black-bear-outreach-project/status-management/; accessed on 12 May 2018). In China, government compensation and commercial insurance are important methods to mitigate HBCs, and commercial insurance is more popular in practices. On the one hand, the commercial insurance covers not only livestock losses caused by wild animals but also livestock losses caused by natural disasters and diseases while the government compensation is only for livestock losses caused by wild animals. On the other hand, commercial insurance has a higher evidence collection efficiency and higher compensation amount [38].

Existing mitigation measures of HBCs have alleviated negative interactions. However, the development of these measures remains at the technical level of the measures themselves, lacking an analysis of local realities and making it difficult to promote globally. For example, electric fencing needs a continuous and stable power supply as well as regular professional maintenance, which is difficult in areas without electricity lacking solar energy resources. Hunting is the quickest way to mitigate HBCs, but hunting can be controversial and unacceptable to the public, especially in areas where Buddhism is practiced [36]. Although bear spray has a great deterrent effect on bears, some countries prohibit the use of spray because it contains capsaicin [39].

**Figure 1:**
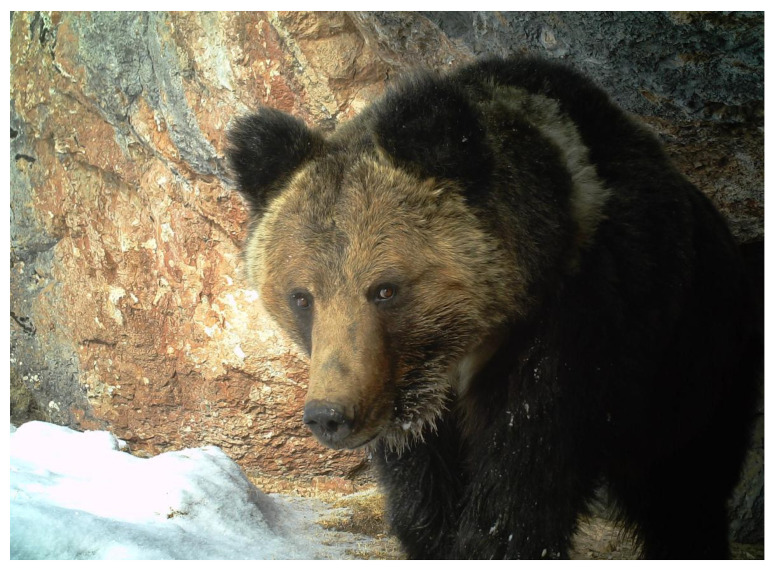
Tibetan brown bear (*Ursus arctos pruinosus*) captured by a camera trap in Sanjiangyuan National Park, China.

The Tibetan brown bear (*U. a. pruinosis*) (Figure 1), an umbrella species for biodiversity conservation on the Qinghai-Tibetan Plateau (QTP), is important for maintaining the health and stability of the ecosystems it inhabits. In recent years, HBCs have intensified in the Sanjiangyuan Region in Qinghai Province, China, decreasing the tolerance of local herders towards the species and seriously affecting the motivation of local communities to protect brown bears and other wildlife, with retaliatory killing posing a threat to their survival [1]. Timely development of effective measures and countermeasures for mitigating HBCs is crucial to protect brown bears. The mitigation or resolution of HBC issues is beneficial to both the promotion of people’s livelihoods and the conservation of brown bears on the QTP. At present, there is still a lack of research on the mitigation measures of HBCs on the QTP. This study combined field surveys, semi-structured interviews, and HBC seminars to understand the effectiveness of current mitigation measures and to propose potential mitigation measures in the hinterland of the QTP. This work proposed targeted mitigation measures for HBCs taking into account existing HBC management practices in China and abroad, and the unique geographical environment, laws and regulations, folk culture, and religious beliefs of local regions. Although this study was limited to a single species on the QTP, the results herein are useful for drafting national-level wildlife conservation policies, compensation programs for wildlife damage, and natural resource conservation regulations.

## 2. Materials and Methods

### 2.1. Study Area

**Figure 2:**
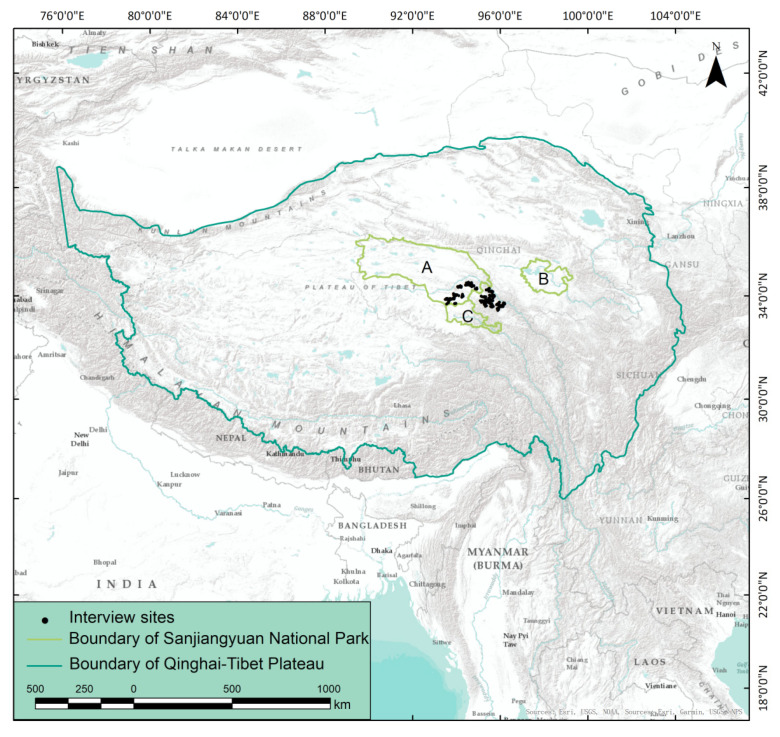
Interview locations in Zhiduo County, Qinghai-Tibetan Plateau, China. A refers to the Yangtze River Zone of Sanjiangyuan National Park (SNP); B refers to the Yellow River Zone of SNP; C refers to the Lancang River Zone of SNP.

The Sanjiangyuan (Source of Three Rivers) Region is an area of the Qinghai-Tibetan Plateau (QTP) in Qinghai Province, China, which contains the headwaters of three great rivers of Asia: the Yangtze River, the Yellow River, and the Lancang River (Figure 2). Its unique plateau ecosystem maintains a large area of alpine swamp meadow and natural habitat of wildlife [40]. The Sanjiangyuan Region is rich in wildlife resources. The representative animals here include snow leopard (*Panthera uncia*), Pallas’s cat (*Otocolobus manul*), Tibetan brown bear, Tibetan wild donkey (*Equus kiang*), and Tibetan antelope (*Pantholops hodgsonii*). Zhiduo County is located in the west of the Sanjiangyuan region, with a total area of about 39,000 km^2^ (excluding the Hoh Xil area). The average altitude of Zhiduo is 4500 m and the area is characterized by high elevations, complex topography, harsh weather, and limited roadways [40]. The weather is typically dry and cold, with an annual average temperature of −0.8 °C and an annual average precipitation of 419 mm [41]. Due to its unique environmental and climatic conditions, alpine meadow is the main vegetation type in the study area. Zhiduo has 68 pastoral communities spread across five townships (Lixin, Zhiqu, Duocai, Zhahe, and Suojia) and one town (Jiajiboluo) with approximately 48,480 permanent inhabitants. Tibetans account for more than 98% of the residents, with the remainder being Han, Hui people, and others (statistical data from 2020; data source: People’s Government of Zhiduo County; http://www.zhiduo.gov.cn, accessed on 5 May 2021). Among them, Suojia and Zhahe are encompassed by the Yangtze River Zone of Sanjiangyuan National Park (SNP) (Figure 2).

### 2.2. Data Collection

#### 2.2.1. Semi-Structured Interviews

Various interview methods can be selected for different subjects, contents, and purposes, including structured interviews, semi-structured interviews, and unstructured interviews [7]. Semi-structured interviews take into account the information quality and questionnaire efficiency, and involve both structured and open questions. As such, this study used semi-structured interviews to investigate HBCs in the area (Supplementary Material). Interviews of herders serving as the head of the household were conducted in Zhiduo County in July and August 2019. Respondents were asked about the types and effectiveness of HBC mitigation measures. Meanwhile, respondents were asked whether they reported HBC cases, the success rate of case loss assessments, compensation rates, and their satisfaction with compensation to understand the problems existing in the current compensation scheme for bear damage. We had two investigation groups to conduct the interviews, and two local guides served as translators from Mandarin Chinese to Tibetan. A total of 312 households were successfully surveyed, with 10 to 20 households surveyed by each pastoral committee (Figure 2).

#### 2.2.2. HBC Seminar

On 12 May 2018, we invited experts, government officials, and representative stakeholders from the Sanjiangyuan National Park (SNP; located in Xining city, Qinghai Province, China), Northwest Institute of Plateau Biology, Chinese Academy of Sciences, IUCN (International Union for Conservation of Nature) Bear Specialist Group, GEF (Global Environmental Facility), Shan Shui (a Chinese NGO), and pastoral committees to participate in an HBC seminar by the SNP Administration. The topic of the seminar was to discuss what kinds of international prevention measures were suitable for alleviating HBCs in the Sanjiangyuan region. In the early stage of the seminar, we invited three experts from the IUCN Bear Specialist Group to conduct a pilot survey in Suojia village in Zhiduo County, and put forward 11 potential HBC mitigation options for the Sanjiangyuan Region based on that field survey and practical experience in other countries (Table 1). At the seminar, we first introduced the current standing of HBCs, the special geographical environment, and the local culture and religion of the Sanjiangyuan Region, and discussed the advantages and disadvantages of the 11 potential solutions. Participants evaluated the potential effectiveness of each solution by voting. If a measure received more than a 50% support rate, it was considered a potential mitigation measure.

## 3. Results

### 3.1. Socio-Demographic Information of Respondents

The respondents were all Tibetan, of whom 74% were male and 26% were female. Most were under 50 years old, with the youngest respondent being 22 years old and the oldest respondent 67. About 56% of the respondents had received a primary school education or below, and only about 5% of the respondents had received a university education or above. About 95% of the respondents were herders, and about 10% of the respondents were park rangers. The remaining respondents were government employees from the government of Zhiduo County and the Management Committee of the Yangtze River Source (Table 2).

### 3.2. Evaluation of Mitigation Measures and Effectiveness

#### 3.2.1. Mitigation Measures

**Figure 3:**
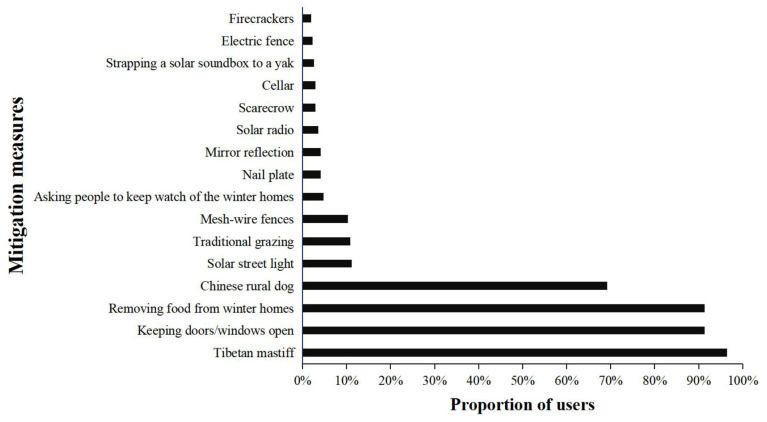
Proportion of users with different bear prevention measures.

Up to 96.47% (*n* = 301) of the respondents used a variety of HBC mitigation measures simultaneously while 3.53% (*n* = 11) of the respondents did not take any measures due to only owning a few livestock and living in densely populated areas. Among the 16 HBC mitigation measures (Figure 3), Tibetan mastiff (guardian dogs) were the most popular (*n* = 301, 96.47%), and 69.23% (*n* = 216) of the respondents kept a Chinese rural dog. To prevent brown bears from breaking into winter homes and damaging supplies, 91.35% (*n* = 285) of the respondents transferred all winter supplies to the summer pasture during the grazing transition and kept the doors and windows open to prevent damage to them. To protect free-ranging livestock, 10.9% (*n* = 34) of the respondents used traditional grazing (all-day livestock guarding) instead of semi-traditional grazing (unguarded during the day and driving livestock back into fenced enclosures before dark). To protect winter homes, 10.26% (*n* = 32) of respondents bought mesh-wire fences, 4.81% (*n* = 15) of the respondents asked people to keep watch of their winter homes, 4.17% (*n* = 13) of the respondents used nail plates, and 4.17% (*n* = 13) of the respondents used mirror reflection to drive away brown bears. During summer grazing months, 3.53% (*n* = 11) of the respondents played 24-h solar radios in unattended settlements, creating the illusion that there were people in the houses. A few respondents used cellars (*n* = 9, 2.88%), scarecrows (*n* = 9, 2.88%), solar soundboxes (*n* = 8, 2.56%), electric fences (*n* = 7, 2.24%), and firecrackers (*n* = 6, 1.92%) to mitigate HBCs.

#### 3.2.2. Effectiveness Assessment

According to the assessment, those who used solar soundboxes strapped to yak (*n* = 8) perceived that it was 100% effective. Respondents indicated that traditional grazing methods (efficiency = 88.24%) were effective at protecting free-range livestock. Removing food from winter homes (efficiency = 87.37%) and asking people to keep watch of the winter homes (efficiency = 86.67%) were effective at protecting food and houses. During the field survey, this study found that 35 families had installed solar street lights near their settlements. Herders indicated that the original purpose of solar street lights was for the convenience of livestock management in the evening. However, it was later found that solar street lights had a certain deterrent effect on wolves (*Canis lupus*), snow leopards, and brown bears (efficiency = 80%). Tibetan mastiff and Chinese rural dogs worked marginally to mitigate HBCs. Some herders kept Tibetan mastiff and Chinese rural dogs simultaneously, which was due to the fact that while Tibetan mastiffs are better adapted to cold and hypoxic environments than Chinese rural dogs, Chinese rural dogs are more sensitive and vigilant. In practice, scarecrows, cellar, keeping doors and windows open, solar radios, and mesh-wire fences were essentially ineffective (Table 3).

### 3.3. Compensation Status

Up to 95.51% (*n* = 298) of the respondents experienced livestock loss in 2018. Among them, 81.41% (*n* = 254) of the respondents reported these cases to the local wildlife management agency. Of the reported cases, 192 herders received compensation while the other 62 herders did not receive any compensation due to a failure of case determination. The success rate of the case determination of livestock loss was about 75.59%. Although 88.46% (*n* = 276) of the respondents experienced house break-ins caused by brown bears in 2018, only 84 herders reported such cases to the local wildlife management agency, and 21 herders finally received compensation. The success rate of the case determination of house break-ins was approximately 25%. Respondents indicated that specialists would be assigned to collect wildlife damage evidence. If case determination succeeded, herders would receive compensation based on economic value. For instance, the compensation amount for a yak was between 150 to 300 USD and between 90 to 130 USD for a sheep. If the case determination failed, no compensation could be obtained.

Up to 71.79% (*n* = 224) of the respondents were dissatisfied with the current compensation scheme for wildlife damage. The main reason was that most home damage caused by brown bears was not compensated, and that herders and the wildlife management agency could not reach an agreement on compensation amounts (*n* = 97, 43.3%). For livestock losses, herders received only about half of the market value for the animal. Other reasons for dissatisfaction included insufficient compensation amounts (*n* = 56, 25%), complex compensation claims (*n* = 38, 16.96%), delay in payment (*n* = 21, 9.38%), and others (*n* = 12, 5.36%). The respondents expressed a desire for case determination and compensation claim procedures to be simplified, compensation amounts to be increased, and compensation schemes for house break-ins specifically to be improved. At present, commercial insurance has been piloted in some areas of Zhiduo County, mainly for livestock. The insurance covered not only livestock losses caused by wild animals but also livestock losses caused by natural disaster and disease. Government compensation was only for livestock losses caused by wild animals, and the amount of compensation paid by insurance companies was higher than that of government compensation.

### 3.4. Potential Mitigation Strategies and Research Options

At the HBC seminar following the field survey, we posed a matrix of potential actions to help rectify existing issues (Table 4). Results showed that effective measures for protecting winter homes were electric fences and mesh-wire fences, among which electric fences were preferred. Effective measures to protect food were electric fences, mesh-wire fences, steel bins, community bins, and elevated platforms, among which electric fences were still the most preferred measure. The most effective measure to protect people was bear spray, but this measure was not the top priority chosen because the Chinese government prohibits citizens from using spray with capsaicin. The most effective measures to protect penned livestock were electric fences and mesh-wire fences. Diversionary feeding may work to help protect livestock, but considering the high cost of diversionary feeding, it was not the preferred measure to protect houses, people, and livestock. However, all participants assumed that this measure could fundamentally solve the problem of HBCs. In addition, the participants all stated that diversionary feeding could provide a direction for later research on HBCs.

## 4. Discussion

HBCs continue to increase, despite herders in the Sanjiangyuan Region taking multi-pronged measures to prevent damage caused by bears [51]. While few herders are currently using strap solar soundboxes to protect free-ranging livestock, this appears to be the most effective measure. However, the effectiveness of this measure needs to be further verified and evaluated after increasing sample sizes. The most effective measure to protect herders’ winter homes was to have other people keep watch. However, the labor force in the Sanjiangyuan Region is decreasing year by year (SNP officers, personal communication). Therefore, it is not practical to rely on people to keep watch of winter homes. Scarecrows and radios were worse in preventing bears from invading winter homes because brown bears no longer feared such measures when they became familiar with these tactics. Brown bears have a keen sense of smell and can easily find cellars where herders store their food. The earthen structure of the cellar was not enough to provide protection and bears easily breached this barrier. Keeping the doors and windows open somewhat reduced the probability of structural damage to the houses by brown bears. However, the daily supplies and furniture inside the houses still were damaged.

Traditional bear control measures were largely ineffective. Barrier fences and electric fences have failed due to improper installation. Since electricity is not yet available in most areas of the Sanjiangyuan region, herders typically use wire fences to protect their homes and livestock. It was reported that local wire fences were installed without considering hardened fence floors, thus leading to the failure of some fences for prevention and control (IUCN Bear Specialist Group, personal communication). For example, the United Nations Development Programme (UNDP) and the Global Environment Facility (GEF) installed 239 sets of bear control fences and 4 sets of livestock inoculation fences in the Sanjiangyuan Region from 2012 to 2017. Because the installation did not harden the entry area of the fence’s fixed stakes, brown bears entered the houses and livestock pens by digging holes from the bottom of the fences, thus leading to their failure. Electric fences are an internationally recognized mitigation measure of HBCs. It has played an important role not only to deter brown bears from entering and destroying homes but also to correct their intrusive behavior [24,32]. However, the existing mobile pulse electric fence in the Sanjiangyuan Region did not properly function. Most electric fence users reported that the electric shock was not strong enough, which may be due to defects in the installation method, rather than a failure of the prevention and control technology. The soil in the Sanjiangyuan region is dry, and electric fences have poor electrical conductivity. It is difficult to form a connected electrical circuit, which causes the high-voltage generator to not work properly. Electric fences rely on ground wires and ground stakes to complete the circuit loop. Dry soil can reduce the sensitivity of high-voltage pulse valves.

In general, herders are reluctant to report minor property damage caused by wildlife [50,51]. However, once damage exceeds their acceptance level, wildlife may face the threat of retaliatory killing. Therefore, compensation for wildlife damage is of great importance to people who live in and outside protected areas and suffer frequent damage from wildlife. At present, the complex case evaluation process, small amount of compensation, delay in payment, and lack of appropriate rules for fixing damage due to brown bears in the Sanjiangyuan Region have led to 71.79% of the respondents being dissatisfied with the current compensation scheme. In the field survey, we found that most herders were not compensated for home damages due to the low success rate of house break-in assessments, and livestock losses were only compensated for about half of their market value. Wildlife management agencies should improve the compensation program for damage caused by wildlife. In particular, it is urgent to develop a reasonable compensation plan for house-break-ins caused by brown bears to minimize the property loss of herders and enhance their tolerance of wildlife.

To protect brown bears, reduce HBCs, and protect the authenticity and integrity of the natural ecosystem in the SNP, the local government should expand the electric fencing pilot area [38]. Simultaneously, an expert team should be invited to improve and update the prevention and control technology of electric fences. The number of ground wires and the depth of the wires into the ground should be adjusted according to differences in the soil dryness and humidity, and a team of local herders or park rangers should be trained to maintain electric fences. In addition, at the HBC seminar, everyone agreed that bear spray is an effective defense against brown bear attacks. It is suggested that a pilot program of bear spray should be conducted in high-risk areas of HBCs, and herders living in high-risk areas should be allowed to use it legally during certain seasons [40]. Bear spray is the last line of defense to safeguard the lives of herders. It has also been an important tool in rebuilding the fear of humans in brown bears after policy changes made by China in 1996 banned firearms and fully eradicated them on the QTP in 2000.

Currently, most herders in the Sanjiangyuan Region no longer store food in their unattended settlements. However, brown bears still habitually visit settlements in search of livestock carcasses and food waste [1]. To reduce the frequency of brown bears foraging in herders’ living areas and to change their foraging behavior, it is suggested that local herders change some of their living habits. Specifically, herders need to move dead livestock away from their living areas. If a large number of livestock die due to disease, the local government should cooperate with the veterinary station for special treatment of livestock carcasses. In addition, the local government should organize specially assigned persons for timely and centralized disposal of herder household garbage. At the HBC seminar, most experts agreed that diversionary feeding could change bear foraging behavior. Therefore, the wildlife management agencies need to establish some diversionary feeding stations for brown bears at suitable locations to reduce the frequency of bears visiting herder living areas in search of food.

Wildlife agencies often implement compensation programs to mitigate human–carnivore conflicts emerging from damage-related losses. Compensation programs present opportunities for pastoral communities to establish close relationships with the national park, engendering trust in authority that can improve attitudes toward conservation [39]. Nevertheless, compensation programs can sometimes further motivate negative attitudes and can be a source of conflict over large carnivore management. In the field survey, we found that local herders were dissatisfied with the current compensation program because of the low efficiency of case determination. Results suggest that wildlife management agencies should improve the efficiency of case determination, simplify claim procedures, increase compensation amounts, and shorten the compensation cycle. However, only one compensation program cannot fully resolve bear damages. In conjunction with the current compensation program, the local government should purchase insurance for herders’ properties to supplement compensation from the destruction of homes and loss of livestock. High-, medium-, and low-grade insurance should be purchased in accordance to high-, medium-, or low-risk areas of HBCs where herders are living. To further improve herder livelihoods and enhance their tolerance of wildlife, this study suggests that the local government improve the ecological grassland award mechanism to protect herder interests. Moreover, herder income patterns should be changed by shifting the focus of their livelihoods to better economic practices. For example, encouraging herders to join ecological public service jobs and develop brown bear-based ecotourism to narrow the gap between limited grazing income and high bear damage prevention costs. Finally, it is suggested that herders are encouraged to participate in wildlife management, and establish friendly relationships between managers, biodiversity conservationists, and stakeholders to enhance motivation for wildlife conservation.

## 5. Conclusions

Since the early 21st century, HBCs have continued to increase, despite people taking multi-pronged measures to prevent damage caused by bears [50,51]. The special geographical environment and folk culture of the QTP have made the study of brown bear ecology and human–bear interactions relatively scarce. HBCs in the Sanjiangyuan Region continue to break through the tolerance of local herders, seriously affecting the motivation of local communities to protect brown bears and other wildlife, and retaliatory killing poses a threat to the survival of brown bears [50]. The results showed that although herders have adopted multi-pronged mitigation measures, incidents of HBCs were still occurring and continued to climb due to improper installation of mitigation measures and limitations of the measures themselves, resulting in unsatisfactory measures. Based on the current characteristics of HBCs, the local geographical environment, and local culture and customs, this study proposed some potential mitigation measures suitable for the Sanjiangyuan Region, such as improving the prevention and control technology of electric fences, expanding electric fencing pilot areas, installing diversionary feeders, and introducing bear spray to mitigate HBCs. This study provides a scientific basis for the development of effective measures for mitigating HBCs on the QTP.

## Figures and Tables

**Table 1:** The potential HBC mitigation options for the Sanjiangyuan Region based on the field survey and practical experience of measures in other countries.

Mitigation Options	The Selection Reasons for Mitigation Options	References
Electric fence	Properly designed and constructed electric fences should be affordable and effective at protecting houses (and the food and people inside houses) from bear intrusions and can also protect penned livestock from depredation events.	[24,32]
Barrier fence	Strong barrier fences should be equivalent to electric fences in keeping bears from entering houses or livestock pens, although with reduced capacity for behavioral modification. They are generally more expensive but require less maintenance.	[42,43]
Steel bins	Steel bins have been used in North America for protecting stored foods from bears. Once purchased, they require no maintenance or special training to operate.	[44]
Elevated platform	Elevated platforms have been used to protect foods or other attractants from bears. They function as effectively as bins and have the advantage of being built on site with local materials.	[14]
Shocker	A device is available that shocks bears when they step on it or touch it. Powered by a solar panel, this could be used to retrain bears that enter houses.	[39]
Bear spray	Containing capsaicin, a very hot pepper that is incapacitating when sprayed into the nose and eyes, is a common useful bear deterrent in North America. It effectively protects people in a close encounter with a bear, and may alter the behavior of bears in terms of their boldness and propensity to approach people in the future.	[32,34]
Diversionary feeding	Diversionary feeding is commonly used in some European countries to mitigate bear conflicts. The concept is to provide a readily available food that substitutes the food bears seek in and around human dwellings.	[45]
Guard dogs	Around the world, dogs are commonly used to protect livestock from predator attacks. Better-trained dogs could more effectively protect free-ranging livestock, and possibly help protect herders.	[46,47,48]
Remove bears	Although bears cannot be killed, some bears could be captured and taken out of the population to a captive facility, where they might undergo a retraining program.	[35]
Relocate people	In an extreme case, people subjected to continual bear problems could be relocated, and the bears in the area would no longer have houses to break into.	[49,50]
Stop pika poisoning	Natural food diversity for bears in the SNP is very limited; it stands to reason that stopping the poisoning of pikas would provide more natural food for the bears, which should lessen their desire for human-related foods.	[39]

**Table 2:** Socio-demographic information of respondents.

Characteristics		Inside of the SNP		Outside of the SNP				Total (%)
		Suojia	Zhahe	Duocai	Zhiqu	Lixin	Jiajiboluo	
Number		75	50	60	43	46	38	312 (100)
Gender	Male	49	38	51	37	29	26	230 (73.72)
	Female	26	12	9	6	17	12	82 (26.28)
Age	≤30	14	23	21	12	16	12	98 (31.41)
	31–50	38	15	34	21	24	20	152 (48.72)
	≥51	23	12	5	10	6	6	62 (19.87)
Education level	≤Elementary	48	35	40	22	19	10	174 (55.77)
	Middle school	21	8	11	14	16	7	77 (24.68)
	High school	4	4	6	5	9	17	45 (14.42)
	≥College	2	3	3	2	2	4	16 (5.13)
Occupation	Herder	73	49	58	41	43	33	297 (95.19)
	Government employee	0	0	0	0	1	4	5 (1.60)
	Park ranger	2	1	2	2	2	1	10 (3.21)
Household size		5.32	5.04	5.15	5.09	5.22	5.08	
Livestock holding per household	Yak	140.41	110.20	105.40	60.87	68.11	45.24	
	Sheep	8.53	6.80	7.33	5.81	4.35	7.37	
	Horse	2.33	1.20	1.87	2.11	1.96	1.24	

**Table 3:** Assessment of the effectiveness for the current mitigation measures.

Prevention Measures	No. of Users	Assessment of Effectiveness		
		Effective	Medium	Ineffective
Keeping doors/windows open	285	31 (10.88%)	38 (13.33%)	216(75.79%)
Removing food from winter homes	285	249 (87.37%)	22 (7.72%)	14 (4.91%)
Tibetan mastiff	301	102 (33.89%)	107 (35.55%)	92 (30.56%)
Chinese rural dog	216	65 (30.09%)	101 (46.76%)	50 (23.15%)
Solar street lights	35	28 (80%)	4 (11.43%)	3 (8.57%)
Traditional grazing	34	30 (88.24%)	3 (8.82%)	1 (2.94%)
Mesh-wire fences	32	5 (15.63%)	4 (12.5%)	23 (71.88%)
Asking people to keep watch of the winter homes	15	13 (86.67%)	2 (13.33%)	0 (0%)
Mirror reflection	13	9 (69.23%)	0 (0%)	4 (30.77%)
Nail plate	13	8 (61.54%)	3 (23.08%)	2 (5.38%)
Solar radio	11	2 (18.18%)	1 (9.09%)	8 (72.73%)
Cellar	9	2 (22.22%)	0 (0%)	7 (77.78%)
Scarecrow	9	1 (11.11%)	0 (0%)	8 (88.89%)
Strapping a solar soundbox to a yak	8	8 (100%)	0 (0%)	0 (0%)
Electric fence	7	3 (42.86%)	2 (28.57%)	2 (28.57%)
Firecrackers	6	3 (50%)	1 (16.67%)	2 (33.33%)

**Table 4:** Participants were presented with a matrix of issues related to bear conflicts (columns) versus potential solutions (rows) and ranked the solutions as likely effective (XX), potentially moderately effective (X), or not likely to address the problem (blank). After this ranking, participants chose the solutions that were most likely to solve the issues (△).

Types	Protect House	Protect Food	Protect People	Retrain or Divert Bears	Protect Penned Livestock	Protect Free-Range Opportunities	Provide Research Opportunities	Recover Natural Food
Electric fence	XX△	XX△	X△	X△	XX△		X△	
Mesh-wire fences	XX	XX	X		XX		X	
Steel bin for house		XX						
Community bin		XX						
Elevated platform		XX						
Shocker in house				X				
Bear spray			XX	X				
Diversionary feeding	X△	X△		X△	X△	X△	XX△	
More/better dogs						X		
Remove bear				XX			XX	
Move people				XX				
Stop pika poisoning				X			XX	XX

## Data Availability

Not applicable.

## Supplementary Materials

The following supporting information can be downloaded at: https://www.mdpi.com/article/10.3390/ani12111422/s1.
