# Supplementary material for: Mitigation Strategies for Human–Tibetan Brown Bear (Ursus arctos pruinosus) Conflicts in the Hinterland of the Qinghai-Tibetan Plateau
Source: Animals (Basel). 2022 May 31;12(11):1422. doi: 10.3390/ani12111422 (PMC9179409; doi:10.3390/ani12111422)
Supplement: Supplementary file 1 [file animals-12-01422-s001.zip › animals-1699305-supplementary.pdf]

Human-wildlife conflict and meadow investigation in Sanjiangyuan

Date________ Translator________ Investigator________

1. Interviewee Info.

1.1County _________ Town_________ Village_________； GPS Location: ____/____ Elevation: ________

1.2 Nationality: □Tibetan □Han Other: ________; Gender: □M □F Name___________ Age____________

1.3 Occupation: □Herder □Governmental officer □Businessman □Other________;

Education: □Uneducated □Primary school □Middle school □Senior high and above

1.4 Income source: □Graze □Caterpillar fungus / herbs □Dogs Other：________________________________

2. House Info.

2.1 Built time：__________

2.2 Live in house season：□Spring □Summer □Autumn □Winter □All year

2.3 Living duration：□＜1 Month □1-3 Month □3-6 Month □6-9 Month □9-12 Month

2.4 House structure：

Door：□Wood □Metal Other______

Window：□Glass＋Wood Frame □Plastic＋Wood Frame □Glass＋Metal Frame □Only Frame Other____；

Wall：□Mud brick □Turf □Air brick＋concrete □Air brick＋concrete＋Tile

2.5 Fence

Fence □None □Barbed Wire □electric fence；Sponsor：□Government □NGO □Personal Other：

If NGO – which one? ___________Built time________；

Fence enclosed the house:_ □Yes □No _______； Fence enclosed the pen:_ □Yes □No

Efficiency：□Yes □ No □Broke Evidence for bear breaking into house? □Yes_______ □No

Evidence for repelling bears? □Yes _______ □No

Maintained by □Family member □ Professional engineer □ NGO □Nobody

2.6 Food storage

Food stored in house: □ Nothing □Butter □Barley □Meat □Flour □Vegetables □Others

Food storage way: _____________

Door open during leaving:_□Yes □No

Food foraged by bears: □ Nothing □Butter □Barley □Meat □Flour □Vegetables □Others

2.7 Mitigation measures

What measures ___________Built time________Efficiency________ □ Effective □Medium □Ineffective

3. Wildlife and bears

3.1 Wildlife you see in summer and winter pasture

Pasture	Grazing time	Wildlife	Remark
□Summer pasture	_M_ D to _M _D		
□Winter pasture	_M_ D to _M _D		

3.2 Brown bear population trends

Outside of conflicts near houses, do you sometimes see bears when you are herding on the summer range?

If so, how many sightings of bears do you make in an average year?

Do you have any feeling about whether the number of bears that you see each year has changed since as far back as you can remember?

Do you think the No. of brown bears changed over the last 10 years？

□Stable □Increase □Decrease □Unknown

How do you know this change?

□See the changes from bear sightings or bear sign (foot prints, scat, etc)

□Changes in the number of homes broken into

□Changes in the number of livestock prayed

□Heard from others Other_________________________

Reasons for brown bears increasing（Open-ended）:

□New no gun policy □National park established □Poaching decreasing □Increase access to human foods

□Othe______________________

Was bears target for hunting before the new gun policy? □Yes □No □Unknown

Reasons for brown bears decreasing:

□ Natural Food decreasing If so, which ones?__________

□Poaching □Poisoning rodents □Other __________ □Do not know

4. Wildlife conflict and herders' perception

4.1 Livestock No.：Yak______ Sheep______ Goat_______ Horse________

Herding dog _______ Types of dog________ Do dogs work for protecting livestocks? □Yes □No

4.2 Grazing conditions

Herder：□Child □Woman □Man □Old man □Hiring □Other_____；No. of grazing people：____

Grazing method ：□Traditional（All day guarding） □Semi-traditional（Driving livestock back in the evening）Other___________

Travel way: □on foot □riding horse □riding motorcycle □driving car

4.3 Changes in yak number over the last 15 years：□Increase by % □Decrease by % □Stable；

Reason：________________________________

4.4 Changes in sheep number over the last 15 years：□Increase by___% □Decrease by___% □Stable；

When did you stop raising sheep? And why?___________

4.5 Brown bear damages

Types of conflicts: A-attack on human B-kill free range livestock C- kill livestock in pen or near house D break into house

Year	Month	Place	Type of conflicts	No. of bears	Facility or livestock Loss	Economic loss	Compensation value

In which year did bear start to break into house? _____________

4.6 People's attitudes toward brown bears

Are you afraid of bears? □Yes □No

Do you like brown bears?

Yes □ Protected species □ It is important to grassland □ It is useful to human

□ Lovely □ Religion Other__________

No □ Dangerous species □ Scared □ Destroy house □ Pray livestock Other______________

Don't care □ Never met bears □No effect to my life □ Do not know about bears □Other

If conflicts were reduced significantly, would that change your attitude towards bears?

Are there any circumstances where an individual bear should be killed?

Are there any circumstances where an individual bear should be captured and taken into captivity?

Do you know what brown bears eat? If so, what? ____

Have you or your family met brown bears？ What were they doing? How far？ How many brown bears？ How did you react？ ____

4.7 Conflicts from other species

	□Wolf	□Snow Leopard	□Lynx	Other_____

What livestock they eat?				
How many livestock they ate last year?				
Depredation season?				
More serious?				

What are the main reasons that affect your life?

□Wolves eat livestock □Snow leopard eat livestock □bears destroy house □snow disaster □disease

□Grassland quality Other：____

What wildlife would you most like to destroy on your grassland? 1____________2____________3______

Why：____

5. Grassland Investigation

5.1 How many years have you lived here? Where did you live before?____

5.2 Here is winter pasture or summer pasture? Why did you build house here：_____________

5.3 The change of your pasture in these 15 years： □Better □Worse □Uncertain □No change □Do not know

Reason____

5.4 What area of pasture do you have?____

5.5 Over the past 15 years, the trend of changes in the number of blue sheep and reasons:

A increase □New gun policy, no poaching □do now know □other________

B decrease □Lack of food □Meteorological disaster □Poaching □Other____

C stable D do not know

5.6 Over the past 15 years, the trend of changes in the number of marmot and reasons:

A increase □New gun policy, no poaching □do now know □other________

B decrease □Lack of food □Meteorological disaster □Poaching □ Government poisoning □Other____

C stable D do not know

5.7 Over the past 15 years, the trend of changes in the number of pika and reasons:

A increase □New gun policy, no poaching □do now know □other________

B decrease □Lack of food □Meteorological disaster □Poaching □ Government poisoning □Other____

C stable D do not know

5.8 Have people ever killed pikas here □Yes. When? Have efficiency？ □Yes □No □No killing

5.9 What wildlife do you see in your pasture or community?

5.10 Summer pasture has more pikas or winter pasture?

5.11 Summer pasture has better quality or winter pasture?
